# Neonatal Graves Disease Masquerading as Hemochromatosis

**DOI:** 10.1210/jcemcr/luae132

**Published:** 2024-07-24

**Authors:** Liesbeth Maggiotto, Steven D Mittelman, Roja Fallah

**Affiliations:** Division of Neonatology, Department of Pediatrics, UCLA David Geffen School of Medicine, Los Angeles, CA 90095, USA; Division of Pediatric Endocrinology; Department of Pediatrics, UCLA David Geffen School of Medicine, Los Angeles, CA 90095, USA; Riley Hospital for Children, Division of Pediatric Endocrinology & Diabetology; Department of Pediatrics, Indiana University School of Medicine, Indianapolis, IN 46202, USA

**Keywords:** neonatal Graves disease, ferritin, liver enzymes, thyroid disease, hemochromatosis

## Abstract

Thyroid autoimmunity is extremely common in the adult population and can affect pregnancy outcomes. Signs in the newborn can range from absent to severe, making the diagnosis easy to miss. We present an interesting case of neonatal Graves disease associated with intrauterine growth restriction, premature delivery, and liver failure with severely high ferritin, thought to be secondary to hemochromatosis. Treatment of the underlying hyperthyroidism caused a rapid resolution of the elevated ferritin and liver failure. This report highlights the importance of considering Graves disease in newborns with liver failure of unknown etiology.

## Introduction

Autoimmune thyroid disease is extremely common, affecting 5% to 15% of women and 1% to 5% of men [1]. Approximately 2% to 4% of pregnant patients are estimated to have thyroid disease, which can have a number of negative impacts on the developing fetus, including stillbirth, preterm delivery, intrauterine growth restriction, preeclampsia, and heart failure (1). Maternal thyroid antibodies can cross the placenta during the second half of the pregnancy. While anti-thyroid peroxidase and anti-thyroglobulin antibodies are not believed to affect the fetal or neonatal thyroid, antibodies that impact signaling through the thyroid stimulating hormone (TSH) receptor can lead to disease in the newborn (2). TSH receptor blocking antibodies (TRAb) can result in transient congenital hypothyroidism, while TSH receptor stimulating antibodies can cause transient neonatal Graves disease (3). Pregnant patients without a known diagnosis of thyroid disease and those who were previously cured can still have TSH receptor blocking or stimulating antibodies that can cross the placenta and cause disease.

The prevalence of transient Graves disease in neonates born to patients with autoimmune thyroid disease is uncertain, varying from 1.5% to 20% in observational cohort studies (3). Neonatal Graves often leads to preterm delivery or in some cases neonatal demise. After birth, usually within the first 10 days of life, infants may exhibit signs in the central nervous system (irritability, sweating, flushing) ophthalmologic (periorbital edema, exophthalmos), and gastroenterological system (weight loss, diarrhea). Less common signs include persistent acrocyanosis, hepatosplenomegaly, thymic enlargement, bruising, petechiae, thrombocytopenia, and liver failure (4). Mortality of untreated severe neonatal Graves disease is 25% (4). It is important to note that the multisystem abnormalities can resemble those seen in infection, inborn errors of metabolism, and other systemic diseases, which may lead to misdiagnosis and delay in appropriate treatment.

We describe a case of neonatal Graves disease with profound metabolic derangements mimicking hemochromatosis, a presentation that has not to our knowledge been previously reported.

## Case Presentation

G.G. was a female infant born at 30 weeks and 4 days of gestation at an outside hospital to a 38-year-old multiparous mother. Mother had a past medical history of asthma, mild gestational diabetes mellitus, and hypothyroidism for which she had been taking Levothyroxine (125 mcg daily). There was no history of liver disease or antithyroid medical treatment. Pregnancy was complicated by preterm labor, and the mother delivered via emergent cesarean section secondary to fetal bradycardia. A vigorous infant with a birth weight of 1.271 kg (2.8 pounds, 34th percentile by Fenton growth chart) was born.

## Diagnostic Assessment

Patient was admitted to the neonatal intensive care unit (NICU) due to prematurity and respiratory distress requiring continuous pulmonary airway pressures. Initial physical exam was notable for respiratory distress, generalized petechiae, questionable bruises, and bloody drainage from the mouth and rectum with coffee-ground emesis. Cord pH was 6.98; however, first capillary blood gas was normal. No goiter or craniosynostosis was appreciated on physical examination. She was started on dextrose 10% with calcium gluconate. Due to concern for infection and disseminated intravascular coagulation (DIC), a complete blood count (Table 1) and blood cultures were drawn. Ampicillin and gentamicin were started, and fresh frozen plasma and platelets were administered empirically.

**Table 1.** Laboratory values prior to transfer for quaternary care

Lab (normal range)	DOL 0	DOL 1	DOL 2	DOL 3	DOL 4	DOL 6	DOL 8
**Hematologic:**							
WBC (5-30 K/μL, 5-30 × 10^9^/L)	**43 K/μL**	**27 K/μL**	**22 K/μL**	**11 K/μL**			
Hematocrit (41-65%)	**58%**	**47%**					
Platelets (150-450 K/μL, 150-450 × 10^9^/L)	**44 K/μL**	**23 K/μL**	**38 K/μL**	**62 K/μL**			
Ferritin (≤400 mcg/L, 6800 nmol/L)				**2050 mcg/L** **34 850 nmol/L**			**7250 mcg/L** **123 250 nmol/L**
Iron (28-140 mcg/dL, 1-5 nmol/L)					**199 mcg/dL** **7.1 nmol/L**		
PT (12-15.5 seconds)					11.4 seconds		12.5 seconds
PTT (25-37 seconds)					25 seconds		32 seconds
INR					1		1.1
Fibrinogen (≤150 mg/dL, 8.3 mmol/L)							**172 mcg/L** **9.5 mmol/L**
**Gastrointestinal:**							
Total bilirubin (≤1 mg/dL, 0.05 mmol/L)		**6 mg/dL** **0.3 mmol/L**	**8.1 mg/dL** **0.44 mmol/L**	**8.9 mg/dL** **0.49 mmol/L**	**7.9 mg/dL** **0.43 mmol/L**	**8.2 mg/dL** **0.45 mmol/L**	**8.1 mg/dL** **0.44 mmol/L**
Direct bilirubin (≤0.2 mg/dL, 0.01 mmol/L)		**4.4 mg/dL** **0.24 mmol/L**	**5.9 mg/dL** **0.3 mmol/L**	**6.8 mg/dL** **0.4 mmol/L**	**6 mg/dL** **0.3 mmol/L**	**6.1 mg/dL** **0.3 mmol/L**	**6.3 mg/dL** **0.3 mmol/L**
AST (≤31 U/L, ≤0.5 ukat/L)				**156 U/L** **2.59 ukat/L**			**605 U/L** **10 ukat/L**
ALT (≤55 U/L, ≤0.9 ukat/L)				**93 U/L** **1.54 ukat/L**			**294 U/L** **4.8 ukat/L**
GGT (≤42 U/L, ≤0.7 ukat/L)				**104 U/L** **1.7 ukat/L**			**817 U/L** **13.5 ukat/L**
Alk phos (≤270 U/L, ≤4.4 ukat/L)				**395 U/L** **6.5 ukat/L**			
Albumin (2.8-4.4 g/dL, 0.4-0.6 mmol/L)				2.6 mg/dL 0.39 mmol/L			
Triglycerides (≤150 mg/dL, <8.3 mmol/L)				**180 mg/dL** **9.9 mmol/L**			
**Endocrine:**							
TSH (0.72-11 μIU/mL, 5–76 pmol/L)				<**0.02 μIU/mL** **0.13 pmol/L**			<**0.02 μIU/mL** **0.13 pmol/L**
Free T4 (0.9-2.3 ng/dL, 0.030.07 nmol/L)					>**7 ng/dL**>**0.24 nmol/L**		>**7 ng/dL**>**0.24 nmol/L**

The normal range for each lab value is shown in parenthesis in the left-hand column. Abnormal values are noted in bold font.

Abbreviations: Alk phos, alkaline phosphatase; ALT, alanine transaminase; AST, aspartate aminotransferase; DOL, day of life; GGT, gamma-glutamyl transferase; INR, international normalized ratio; PT, prothrombin time; PTT, partial thromboplastin time; T4, thyroxine; WBC, white blood cell.

Initial laboratory evaluation revealed leukocytosis (white blood cells 41 000 cells per microliter) and thrombocytopenia (platelets 44 000 cells per microliter) (Table 1). On day of life (DOL) 1, the patient was noted to have jaundice with direct hyperbilirubinemia. Further workup was remarkable for elevation of aspartate transaminase, alanine transaminase, alkaline phosphatase, triglycerides, iron, and an extremely elevated ferritin (5 times the upper limit of normal). Coagulation studies, urine cytomegalovirus polymerase chain reaction, toxoplasma titers, blood cultures, amino acid levels, ammonia, and alpha-1 anti-trypsin were all normal. Head and abdominal ultrasounds revealed no abnormalities.

The patient received antibiotics for 4 days, along with caffeine for apnea of prematurity after a few episodes of apnea. She was noted to be intermittently tachycardic and found to have cardiomegaly on echocardiography. She was gradually weaned to room air and reached full feeds at DOL 7. Newborn screening TSH was reported as normal. Of note, newborn screening only reports TSH levels that are higher than range. When we requested the actual value for TSH through newborn screening, the level was reported as zero, which retrospectively was a falsely normal result. Thyroid function tests resulted a low TSH < 0.02 μIU/mL and elevated free thyroxine (free T4) above 7 ng/dL. At DOL 8, the patient exhibited worsening transaminitis, elevated ferritin, and continued hyperbilirubinemia. Thyroid function tests were reordered, and the patient was transferred to quaternary care to rule out neonatal hemochromocytosis (NH) and hemophagocytic lymphohistiocytosis (HLH).

On transfer, the infant was tachypneic and tachycardic, with worsening liver failure, persistent thrombocytopenia, and a very elevated ferritin level. Clinically, the infant was not bleeding and had no further evidence of coagulopathy. Caffeine was discontinued due to persistent tachycardia. An echocardiogram was normal. The gastroenterology service was consulted for a working diagnosis of NH and recommended to test urine for succinyl acetone to evaluate for tyrosinemia and urine reducing substances to rule out galactosemia, which both resulted negative. Alpha-fetoprotein was elevated at 8089 ng/mL (6713 IU/mL; reference range ≤ 8.8 ng/mL [< 7.3 IU/mL]) but not considered high enough to support the diagnosis of NH. Repeat abdominal ultrasound was normal. Repeat laboratory evaluation on DOL 10 resulted in even higher ferritin at 9567 μg/L (162 639 nmol/L; reference range ≤ 400 mcg/L [< 6800 nmol/L]), and total/direct bilirubin 10.4/7.9 mg/dL (0.57/0.43 mmol/L; reference range ≤ 1 mg/dL [0.05 mmol/L]). Iron elevated to 192 μg/dL (6.9 nmol/L; reference range 28-140 mcg/dL [1-5 nmol/L]) with 87% saturation. Ursodiol was started. Liver and buccal biopsy and magnetic resonance imaging were considered to evaluate for iron deposition, but the hematology/oncology service recommended against this, due to a low suspicion for HLH.

Confirmatory thyroid function tests were consistent with prior results. Endocrinology service was consulted, and recommended checking triiodothyronine (T3) level as well as thyroid stimulating immunoglobulin (TSI) and TRAbs. While hemochromatosis can cause a transient hyperthyroidism due to thyroid gland destruction and release of preformed T4, endocrine service had a high index of suspicion for neonatal Graves disease given maternal history of thyroid disease. The high T3 to T4 ratio was considered to be more consistent with a hyperactive thyroid gland than glandular destruction from hemochromatosis, which would be expected to release primarily fully formed T4.

## Treatment

Methimazole (0.5 mg/kg/day) was initiated on DOL 11, even before thyroid antibody tests confirmed the diagnosis of neonatal Graves disease. Because the patient was stable on room air, afebrile, and not in acute distress, additional treatments such as steroids and potassium iodide drops were not initiated. The baby was tachycardic while on caffeine; thus, as the first intervention for tachycardia, we discontinued caffeine which resulted in resolution of tachycardia—no beta blocker was started.

**Figure 1.**
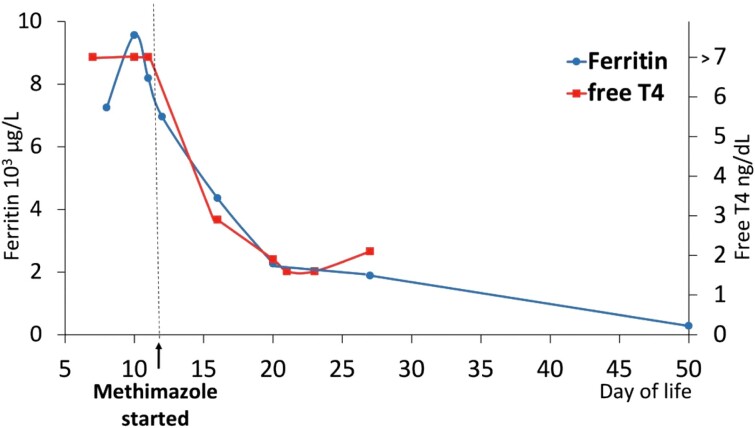
**Ferritin and free T4 test results.** Both Ferritin and free T4 levels continued to improve over the course of time.

An improvement in total T3 and free T4 was seen on day 5 of methimazole treatment (DOL 16), along with a decrease in liver transaminitis and ferritin concentrations. Patient was noted to have positive TSI at 340% (reference range < 130%) and TRAbs 14.07 IU/L (reference range < 1.22 IU/L) confirming the diagnosis of neonatal Graves disease. Methimazole treatment was continued until normalization of T3 and free T4 (Table 2) while liver function tests and ferritin continued to improve (Fig. 1). TSH levels remained suppressed for a prolonged period, as is expected in this condition due to atrophy of TSH-producing cells in the anterior pituitary gland.

**Table 2.** Thyroid function tests at quaternary care facility

Lab (normal range)	DOL 10	DOL 11	DOL 12	DOL 16	DOL20	DOL 23	DOL 27
TSH (0.72-11 μIU/mL, 5–76 pmol/L)	<**0.02 μIU/mL** **0.13 pmol/L**	<**0.02 μIU/mL** **0.13 pmol/L**		<**0.02 μIU/mL** **0.13 pmol/L**	<**0.02 μIU/mL** **0.13 pmol/L**	<**0.02 μIU/mL** **0.13 pmol/L**	<**0.02 μIU/mL** **0.13 pmol/L**
T3, Total (85-185 ng/dL, 2.9-6.4 nmol/L)		>**650 ng/dL**>**22 nmol/L**		**187 ng/dL** **6.4 nmol/L**	**188 ng/dL** **6.5 nmol/L**	151 ng/dL 5.2 nmol/L	176 ng/dL 6.1 nmol/L
Free T4 (0.9-2.3 ng/dL, 0.030.07 nmol/L)	>**7.0 ng/dL** > **0.24 nmol/L**	>**7.0 ng/dL** >**0.24 nmol/L**		**2.9 ng/dL** **0.1 nmol/L**	1.9 ng/dL 0.06 nmol/L	1.6 ng/dL 0.05 nmol/L	2.1 ng/dL 0.07 nmol/L
T4, total (6.4-13.3 mcg/dL, 83-172 nmol/L)						13.1 μg/dL 169 nmol/L	
TSI (<122%)			**340%**				
TRAb (<1.75 IU/L)			**14.07 IU/L**				

The normal range for each lab value is shown in parenthesis in the left-hand column. Abnormal values are noted in bold font.

Abbreviations: T3, triiodothyronine; T4, thyroxine; TRAb, thyrotropin receptor antibody; TSH, thyroid stimulating hormone; TSI, thyroid stimulating immunoglobulin.

## Outcome and Follow-Up

The infant was discharged home at 1 month of age with follow-up appointments with ophthalmology, endocrinology and high-risk-infant follow-up clinic (HRIF). Three weeks after discharge, ferritin levels completely normalized. Methimazole was weaned off at around 10 weeks of age. Baileys III composite score at 4 months old was average in all categories.

## Discussion

We present an interesting case of a premature infant presenting with profound transaminitis, direct hyperbilirubinemia, thrombocytopenia, severely elevated ferritin levels, and resolved coagulopathy, all of which appear to be secondary to neonatal Graves disease. The presentation was confusing due to multiple potential causes of liver damage and the extremely high ferritin level.

The initial presentation of acidosis in the setting of emergent cesarean delivery raised the possibility of hypoxic injury to multiple organs, including the liver. However, the infant's first blood gas was normal, and the normal neurologic exam argues against significant hypoxic injury. Further, hypoxic ischemic injury would be expected to cause elevated liver enzymes immediately after birth followed by a slow resolution. In the present case, the opposite pattern was observed, with gradually worsening liver failure that peaked at DOL 10 to 11.

The severity of the presentation and elevation of liver enzymes and ferritin led to a working diagnosis of HLH or NH. Infants with HLH display severe hyperinflammation caused by uncontrolled proliferation of activated lymphocytes and macrophages. These infants are usually very sick, and mortality is up to 50% (5). Our infant was on room air and full feeds which argued against this diagnosis. NH, also known as gestational alloimmune liver disease, is one of the most common causes of liver failure in the neonatal period. Etiology is due to maternal exposure to fetal hepatocyte-antigens, resulting in production of IgG antibodies that cross the placenta, leading to cirrhosis (50%), liver failure, and hemosiderosis. These infants typically present with severe coagulopathy (average INR > 4) (5), hypoglycemia, and in 10% to 15% of cases, thrombocytopenia. The success of correcting the coagulopathy in our patient by only a few blood transfusions made this diagnosis less likely.

Graves hyperthyroidism is known to cause multisystem effects including cardiac, neurological, ophthalmological, and gastrointestinal disease. Thus, this disease can mimic a number of other diseases of the neonate, including sepsis, TORCH infections, inborn errors of metabolism, bile acid synthesis defect, and Alagille syndrome. Iron homeostasis is also affected by Graves disease, resembling a classical acute phase reaction with rising ferritin and hepcidin. The pathophysiology of this phenomenon may be due to direct stimulation of ferritin production, as ferritin expression in cell culture models has been shown to increase in response to T3 (6). Animal studies in the early 1990s showed that liver iron can be increased by both hypo- and hyperthyroidism (7). However, hypothyroidism decreases while hyperthyroidism increases liver ferritin synthesis in rats. Hyperthyroidism also resulted in elevated transaminases in this rat model (7). A study by Takamatsu et al describe serum ferritin as a marker of thyroid hormone action on peripheral tissues (8). Patients with hyperthyroidism due to Graves disease show a decreasing serum ferritin level as they became euthyroid during antithyroid drug therapy (8, 9).

While previous case reports have described neonatal Graves disease presenting with liver cholestasis, DIC with coagulopathy, or mild-to-moderate elevation in ferritin level, this is the first report to our knowledge to show such a severe rise in ferritin mimicking hemochromatosis. Our patient's newborn screen resulted as negative for congenital hypothyroidism. California and many other states measure TSH, and only report elevated results. Some states measure thyroxine levels directly, but these only report low levels. Neither of these strategies would pick up neonatal Graves disease. Interestingly, although the screen was reported as normal, the measured level was internally reported as 0, which was communicated to us only on request. In hindsight, this level could have been requested earlier and may have helped establish the diagnosis earlier.

Although neonatal Graves is usually self-limiting, due to the slow clearance of circulating antibodies over time, it can be life threatening, leading to liver failure and death if not recognized and treated early. There is not always a clear history of autoimmune thyroid disease in the mother and it can therefore be easily missed in the neonate. This report highlights the importance of considering Graves disease in newborns with liver failure of unknown etiology.

## Learning Points

- There is not always a clear history of hyperthyroidism in mothers of infants with neonatal Graves disease.
- Neonatal Graves is known to be a multisystem disease and may mimic a number of other diseases of the neonate, including sepsis, TORCH infections, inborn errors of metabolism, bile acid synthesis defect, and Alagille syndrome.
- Graves hyperthyroidism presents itself in a variety of ways, including liver failure, coagulopathy, and, in this case, severely elevated ferritin levels mimicking hemochromatosis.
- This case illustrates that iron homeostasis is also affected by Graves disease, resembling a classical acute phase reaction with rising ferritin and hepcidin.
- A normal newborn screening does not necessarily mean normal thyroid function. Understanding your state's newborn screening practices can help in interpreting negative screens.

## Contributors

L.M. was involved in the neonatal intensive care unit (NICU) care as well as writing the Case Presentation and Outcome and Follow-Up. S.M. was involved in the diagnosis and management of the patient and revising the manuscript. R.F. was involved in the diagnosis, management of the patient, writing the Introduction and Discussion, revising the manuscript, and submission. All authors reviewed and approved the final draft.

## Data Availability

Original data generated and analyzed during this study are included in this published article. The authors declare that they have no relevant or material financial interests that relate to this case report.

## Abbreviations

DIC: disseminated intravascular coagulation
DOL: day of life
HLH: hemophagocytic lymphohistiocytosis
NH: neonatal hemochromocytosis
T3: triiodothyronine
T4: thyroxine
TRAb: thyrotropin (TSH) receptor antibody
TSH: thyrotropin (thyroid stimulating hormone)
TSI: thyroid stimulating immunoglobulin
